# A strain comparison of *Campylobacter* isolated from retail poultry and human clinical cases in Atlantic Canada

**DOI:** 10.1371/journal.pone.0215928

**Published:** 2019-05-08

**Authors:** Lisa M. Hodges, Catherine D. Carrillo, Jacqueline P. Upham, Antonela Borza, Mikaela Eisebraun, Robyn Kenwell, Steven K. Mutschall, David Haldane, Emily Schleihauf, Eduardo N. Taboada

**Affiliations:** 1 Canadian Food Inspection Agency, Dartmouth, Nova Scotia, Canada; 2 Canadian Food Inspection Agency, Ottawa Laboratory (Carling), Ottawa, Ontario, Canada; 3 Bureau of Microbial Hazards, Food Directorate, Health Canada, Ottawa, Ontario, Canada; 4 National Microbiology Laboratory at Lethbridge, Public Health Agency of Canada, Lethbridge, Alberta, Canada; 5 Queen Elizabeth II Health Sciences Centre, Mackenzie Building, Halifax, Nova Scotia; 6 Public Health Agency of Canada, Ottawa, Ontario, Canada; Cornell University, UNITED STATES

## Abstract

*Campylobacter* is the leading cause of food-borne bacterial disease in Canada and many developed countries. One of the most common sources of human campylobacteriosis is considered to be the consumption or handling of raw or undercooked poultry. To date, few Canadian studies have investigated both the prevalence of *Campylobacter* on retail poultry and its potential impact on human clinical cases. The objective of this study was to evaluate the prevalence of *Campylobacter* spp. at the retail level and the correlation between subtypes recovered from chicken and those recovered from human clinical cases within the province of Nova Scotia, Canada. From this study 354 human clinical isolates were obtained from provincial hospital laboratories and a total of 480 packages of raw poultry cuts were sampled from retail outlets, yielding 312 isolates (65%), of all which were subtyped using comparative genomic fingerprinting (CGF). Of the 312 chicken isolates, the majority of isolates were *C*. *jejuni* (91.7%), followed by *C*. *coli* (7.7%) and *C*. *lari* (0.6%). Using CGF to subtype *C*. *jejuni* and *C*. *coli* isolates, 99 and 152 subtypes were recovered from chicken and clinical cases, respectively. The most prevalent human and chicken subtypes found in NS are similar to those observed nationally; indicating that the *Campylobacter* from this study appear to reflect of the profile of *Campylobacter* subtypes circulating nationally. Of the subtypes observed, only 36 subtypes were common between the two groups, however, these subtypes represented 48.3% of the clinical isolates collected. The findings from this study provides evidence that in Nova Scotia, retail poultry can act as a reservoir for *Campylobacter* subtypes that have been implicated in human illness.

## Introduction

Amongst developed countries, including Canada and the USA, *Campylobacter* spp. are one of the most common causes of food-borne bacterial gastroenteritis reported each year [1–3]. In Canada, 20,450 cases of campylobacteriosis were reported between 2012 and 2013, an average of approximately 28.5 cases/ 100,000 population (NDO). While campylobacteriosis is typically self-limiting, the severity of the illness can range from asymptomatic to severe enteritis. Typical symptoms include diarrhea, abdominal pain, fever, anorexia and headache, however, in some cases, infections can lead to hospitalizations, post-infection sequelae and death [4]. In addition, infection by *Campylobacter jejuni* is also considered to be the most common antecedent infection of Guillan-Barré syndrome (GBS), an autoimmune disease resulting in paralysis and even death [2,5,6]. Other sequelae include reactive arthritis and inflammatory bowel disease [7]. *C*. *jejuni* is isolated from the majority of human cases of campylobacteriosis (> 90% of cases), followed by *C*. *coli* and *C*. *lari* [8].

The vast majority of reported human infections are considered to be sporadic, although outbreaks are sometimes reported [9–11]. Moreover, potential case clusters can be identified when enhanced molecular surveillance is applied [12]. Although direct attribution of a food or water source to a *Campylobacter* infection has not typically been possible, risk assessments, comparative exposure assessments, case studies and retrospective studies have been used to evaluate the most likely sources of *Campylobacter* infections when no source is available [10,13,14].

Strain typing can be used to identify strains most commonly associated with a specific source (e.g. animal, food or water), which can be used to establish probable links to strains isolated from clinical cases. Methods routinely used for subtyping of *Campylobacter* can vary significantly in the degree of strain resolution that can be achieved and in the ability to infer linkages between illness and source. Commonly used subtyping methods for *C*. *jejuni* and *C*. *coli* have included pulsed field gel electrophoresis (PFGE), restriction fragment length polymorphism of the flagellin gene short region (*flaA* RFLP) and multilocus sequence typing (MLST) [15]. Although these methods have been widely used in research studies aiming to examine the epidemiology of campylobacteriosis, utility in a public health context for providing insights on the possible source of an infection or identifying case clusters from an outbreak can be somewhat limited. For example, it has been reported that MLST may lack discriminatory power among certain strains harbouring significant genetic differences [16]. This can result in large clusters of epidemiologically unrelated isolates, which can make it difficult to gain meaningful information for source tracking [17,18]. PFGE, in contrast, is greatly affected by genetic recombination and high genetic diversity, which can lead to difficulties in clustering closely related isolates, such as those associated with an outbreak [19]. Most recently, a comparative genomic fingerprinting (CGF) method was developed to address these problems. CGF has been found to have a high degree of correlation to MLST typing but with the higher discriminatory power required to differentiate between closely related isolates [17,18]. This method is a high-throughput, multiplex PCR method which identifies the presence or absence of 40 accessory genes of *Campylobacter* to develop a subtype profile. The genes used for typing include both putative genes and genes which have known roles such as in iron acquisition, flagellar modification and capsule and lipooligosaccharide biosynthesis [17]. The CGF method has been extensively used in Canada to characterize isolates generated through a number of large-scale surveillance programs and *ad hoc* research activities [15]. A national database comprising data on over 21,000 isolates collected from human, animal, and environmental sources has been established to facilitate studying the epidemiology of *Campylobacter* strains circulating in Canada [15].

Regardless of the typing method used, the consumption or handling of raw or undercooked poultry has been identified as a major contributor to human campylobacteriosis [10,20,21]. There are currently few studies that have investigated the prevalence of *Campylobacter* spp. on raw poultry products sold at the retail level in Canada [22–25]. Of these studies, none have focused on poultry sold in the Canadian Atlantic provinces. The aims of this study were to i) estimate the prevalence of *Campylobacter* spp. on raw, packaged poultry sold in Nova Scotia, Canada, ii) use CGF as a high-resolution method to subtype isolates recovered from poultry samples and human clinical cases in Nova Scotia, and iii) evaluate the correlation between CGF subtypes recovered from poultry and those known to cause human illness.

## Materials and methods

### Isolation and identification of *Campylobacter* spp.

#### Sample collection

From mid-July to mid-October 2012 (16 weeks), 30 retail packages per week of raw chicken cuts were purchased from multiple large grocery stores in Dartmouth, Nova Scotia, Canada (n = 480). Packages were selected to include as many varieties of cuts, lot numbers and registered meat establishments as possible. The packages purchased included 198 packages of breast cuts, 50 packages of wings, 123 packages of thigh cuts, 34 packages of leg cuts, and 75 packages of drumsticks. The packaged chicken cuts weighed at least 250–500 g to ensure sufficient amount of sample for testing, and were purchased before the ‘best before’ date. Samples were maintained at 2–4°C prior to testing which was initiated within 24 h of samples arriving at the laboratory.

#### Recovery of *Campylobacter* from poultry

Microbiological analysis was conducted as described previously with some modifications [26]. Briefly, each package was wiped with 5% Dettol and aseptically opened with a sterilized blade. Approximately 200–500 g of whole pieces (e.g. 1 breast, 1 leg, 2–3 drumsticks) were aseptically placed in a sterile stomacher bag and 150 ml of Bolton broth (no supplements, Oxoid, Nepean, Canada) was added. Samples were shaken aerobically at 37°C and 100 rpm for 30 min. From this, 100 ml of broth was transferred to a 7 oz Whirlpak round-bottom stomacher bag and 1 ml of antibiotic supplement was added (final concentration of 20 mg/L trimethoprim-HCl (Sigma, Oakville, Canada), 20 mg/L vancomycin (Sigma) and 20 mg/L sodium cefoperazone (Sigma)). Samples were incubated at 42°C under microaerobic conditions (5% O_2_, 10% C_2_O and 85% N_2_), for 48 h.

After 48 h three 15 μL droplets were pipetted onto 47 mm diameter, 0.65 μm pore size Millipore nitrocellulose filters, laid over antibiotic-free *Campylobacter* Agar with Charcoal and Deoxycholate (mCCDA, Oxoid, Nepean, Canada). After 15 min the filter was removed and plates were streaked to disperse bacterial cells for single colonies. Plates were incubated at 37°C for up to 48 h under microaerobic conditions.

From each plate, up to three presumptive *Campylobacter* colonies were streaked onto mCCDA and incubated at 37°C microaerobically for 24–48 h. Pure cultures were checked for characteristic motility and morphology by microscopy. A single presumptive *Campylobacter* colony was inoculated into 3 mL of Brucella broth (Oxoid, Canada) and incubated at 37°C microaerobically for up to 48h. One ml of the broth culture was used for species identification by PCR and 0.5 ml was used for preparing stock cultures to be frozen at -80ºC in 20% glycerol.

#### Collection of clinical isolates

Human *Campylobacter* spp. clinical isolates at the QEII Microbiology Laboratory in Halifax, NS were collected both retrospectively and prospectively from hospitals in Nova Scotia between January, 2012 and March, 2015. Cultures were sent to the laboratory in frozen cryovials or Amies swabs. Isolates were plated onto either antibiotic-free mCCDA or Mueller Hinton blood agar and incubated at 37°C microaerobically for up to 72h. Species identification and frozen stock culture preparations were performed as described below.

#### PCR confirmation

Presumptive *Campylobacter* spp. isolates were confirmed using a multiplex PCR assay as described previously [26,27]. Isolates were determined to be *C*. *jejuni* based on the detection of the hippuricase gene and *C*. *coli* based on the detection of an aspartokinase gene [28], with universal 16S ribosomal RNA gene primers used as an internal amplification control. DNA was extracted from 1 ml of pure culture grown in Brucella broth after at least 24h of growth. Cells were pelleted at 5,800 x g for 10 min and resuspended in 50 μL of lysis buffer (0.25% SDS, 0.05 M NaOH). Lysates were then heated for 8 min at 99°C and 250 μL of 6% Chelex-Resin solution (100–200 mesh, sodium form, Bio-Rad) was added. The lysates were vortexed and heated for an additional 8 min. Lysates were then cooled for 5 min on ice. Cell debris was pelleted by centrifugation at 13,000 x g for 5 min. The supernatant containing the crude bacterial DNA was transferred to a 1.5 ml microcentrifuge tube and was stored at -20°C until required. All PCR reactions were performed in 25 μL volumes, consisting of 12.5μL Gotaq Green master mix (Promega), 1.2 μM of each primer, 2 μL of DNA extract and molecular grade water. PCR cycling parameters were as follows; 95ºC for 10 min, 35 cycles of 95ºC for 30 s, 50ºC for 30 s and 72ºC for 1 min and final elongation of 72ºC for 10 min. PCR products were visualized on a 1.2% agarose flash gel (Lonza). Isolates which were negative for *C*. *jejuni* and *C*. *coli* but had a positive IAC PCR product, were further confirmed using API Campy strips (Biomerieux).

### Subtyping by CGF

#### Comparative genomic fingerprinting

CGF subtypes were generated for each isolate using primer sets and methodology described previously [17]. Briefly, CGF is a high-throughput PCR-based typing method that produces a binary profile for each isolate based on presence or absence of each of 40 target amplicons. Binary profiles were assigned a three-digit CGF subtype based on a nomenclature derived from cluster membership in the Canadian *Campylobacter* CGF database (C3GFdb). Briefly, binary fingerprints were hierarchically clustered using the unweighted pair group method with arithmetic mean (UPGMA) and the simple matching coefficient (i.e. Hamming distance) in BioNumerics (version 6.6, Applied Maths, Austin USA). Clusters were then assigned at three-digit subtype reflective of hierarchical profile similarities such that profiles similar at >90% and <95% level share only the first digit, profiles similar at >95% and <100% level share the first and second digits, and profiles similar at the 100% level share all three digits. Profiles sharing <90% similarity do not share any digits. In cases where novel patterns were obtained, these were assigned a new CGF subtype based on the most similar fingerprint in the database and the hierarchical nomenclature rules outlined above. The C3GFdb, developed and maintained by the National Microbiology Laboratory (Public Health Agency of Canada), comprises a pan-Canadian collection of *Campylobacter* isolates (n = 21,232) from human (n = 4,669), animal (n = 13,345) and environmental (n = 2,980) sources from across Canada, representing 4,935 unique CGF subtypes and obtained from samples collected from 1998 to 2018 through several national/regional surveillance programs and a range of *ad hoc* research projects.

Subtype relationships were visualized using a minimum spanning tree (MST) produced with GrapeTree (version 0.1.0) [29].

#### Source attribution of human clinical cases

Source attribution for Nova Scotia human clinical cases reported to Nova Scotia provincial public health laboratories between January 2012 and March 2015 (n = 354) was investigated using a basic Dutch source attribution model [30]. Under the model, inference on the likely source of human infections is based on the relative frequencies of the strain subtypes among the possible sources and in human cases. For each human clinical case in the dataset, attribution is based on the proportional representation of its subtype among non-human isolates with a matching subtype in the reference database (i.e. C3GFdb). The overall proportion of human clinical cases attributable to the various non-human sources is then computed by aggregating individual proportional attribution estimates across all clinical cases.

### Statistical analysis

Results of the retail poultry sampling was analysed using the GraphPad software QuickCalcs online calculator (www.graphpad.com). A chi-square analysis was used to compare the observed and expected frequencies of positive samples among types of poultry cuts (e.g. breast, drumstick), bone and boneless cuts, skin and skinless cuts, meat establishments and sampling weeks.

## Results

### Prevalence of *Campylobacter* observed in Nova Scotia poultry products

**Fig 1:**
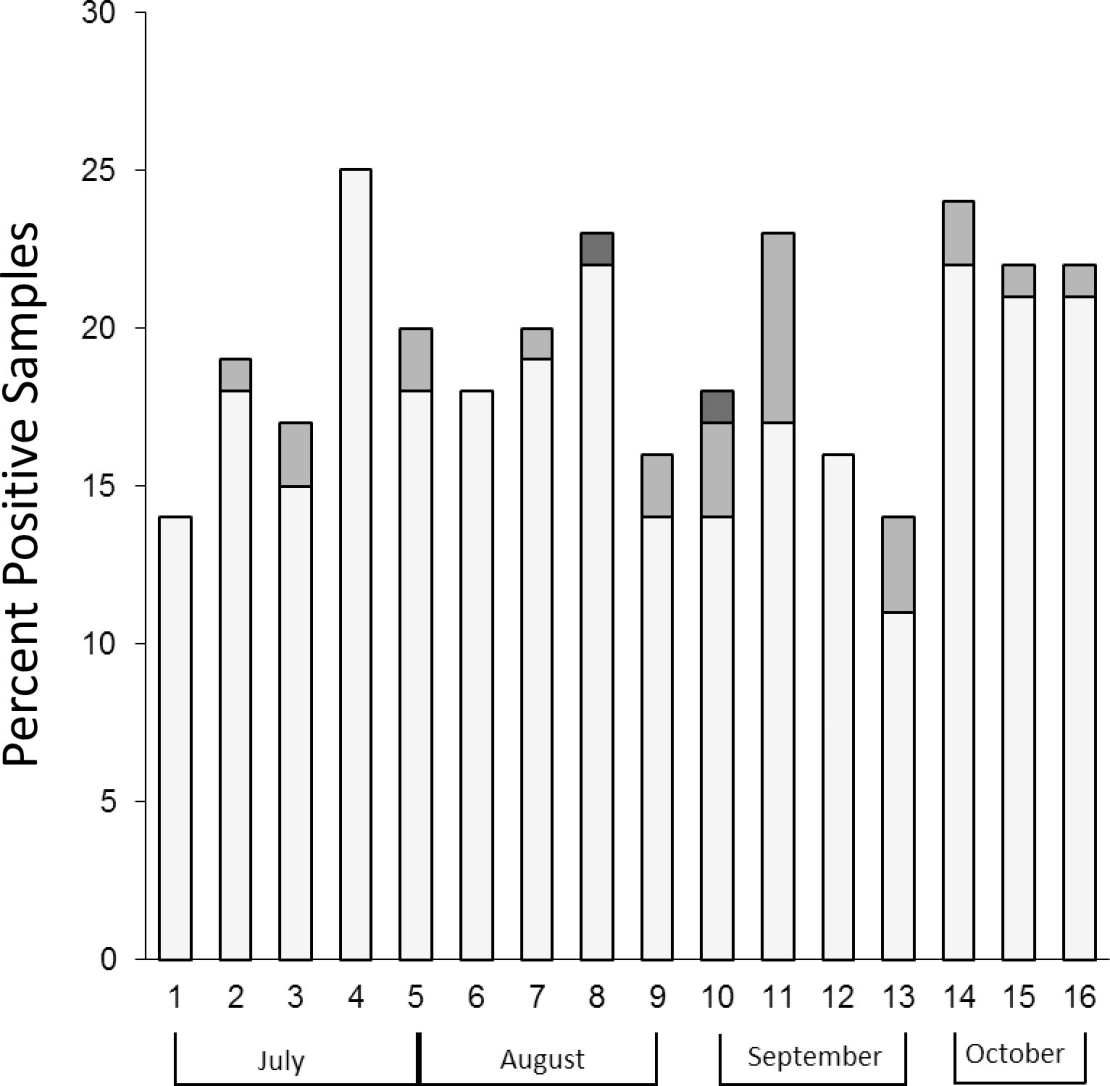
Prevalence of positive samples during the study period. The number of *C*. *jejuni* (light grey), *C*. *coli* (medium grey) and *C*. *lari* (dark grey) isolated each week from 30 packages of retail poultry cuts.

*Campylobacter* spp. was isolated from 312 (65%) of the 480 samples tested during the 16-week study. Of the isolates recovered, *C*. *jejuni* was the predominant species (91.7%), compared to 7.7% of isolates identified as *C*. *coli* and 0.6% isolates identified as *C*. *lari* (Fig 1). The number of samples positive for *Campylobacter* varied from week to week, ranging from 46% to 80%, however, no statistical significance (p > 0.05) was observed between the difference in the number of positive samples recorded weekly, monthly or between the summer (July and August) and fall (September and October) months (Fig 1).

The retail packages tested included 10 federally registered meat establishments sold under several commercial names, all major types of cuts (Table 1), skin-on and skinless cuts (55.4% and 44.6% of all samples, respectively) and bone-in and deboned cuts (52.9% and 47.1% of all samples, respectively). Although thigh cuts were found to have the highest prevalence of *Campylobacter* (73.9%, Table 1), the number of samples positive for *Campylobacter* was not significantly (p > 0.05) influenced by the type of cut, meat establishment, presence of skin or bones.

**Table 1:** The total number (%) of *Campylobacter* spp. recovered from each of the major types of retail poultry cuts tested.

Type of Cut^a^	No. of Samples	Positive (%)	*C*. *jejuni*	*C*. *coli*	*C*. *lari*
Breast	198	123 (62.1)	114 (92.6)	9 (7.3)	0
Drumstick	75	47 (62.6)	43 (91.4)	4 (8.5)	0
Leg	34	22 (64.7)	18 (81.8)	2 (9.1)	2 (9.1)
Thigh	123	91 (73.9)	82 (90.1)	9 (9.9)	0
Wing	50	29 (58)	29 (100)	0	0
Total	480	312 (65)	286 (91.7)	24 (7.7)	2 (0.6)

a. Categories include all types of processing (skin-on and skinless, boned and deboned).

### Comparison of Nova Scotia subtypes to those circulating in the Canadian chicken production chain

Of the isolates recovered from retail chicken samples collected in this study, 292/312 (93.6%) were subtyped by CGF. Overall, 99 CGF subtypes were observed, with the 20 most prevalent chicken subtypes in this study (Table 2) accounting for 57.7% of isolates collected over the sampling period and representing 31.3% to 79.2% of the isolates recovered each week. The two most abundant subtypes (0117.001.001, n = 31 and 0926.002.001, n = 25) were observed in 9 of the 16 weeks of the study and accounted for 17.6% of the isolates recovered. Among the 99 subtypes observed, 17 corresponded to novel fingerprints that had not been previously observed among isolates represented in the C3GFdb. However, these subtypes corresponded to a small number of isolates (n = 20) and included 15 subtypes represented by a single isolate.

**Table 2:** Characteristics of the top 20 CGF subtypes observed among the chicken isolates collected in this study.

CGF subtype	Species	NS Chicken Isolates (n = )	NS Human Isolates (n = )	Overall Rank^1^	Overall Chicken Rank^1^	Overall Human Rank^1^	Non-Human Source Specificity	Source Specificity^2^
0117.001.001	*C*. *jejuni*	31	7	15	4	52	Chicken	100%
0926.002.001	*C*. *jejuni*	25	13	4	2	7	Chicken	81%
0882.005.001	*C*. *jejuni*	15	6	11	6	37	Chicken	90%
0957.001.001	*C*. *jejuni*	11	4	8	7	32	Chicken	79%
0253.004.001	*C*. *jejuni*	10	14	10	5	9	Chicken	98%
0960.007.001	*C*. *jejuni*	10	6	13	9	12	Chicken	78%
0123.001.002	*C*. *jejuni*	9	5	30	12	63	Chicken	100%
0083.001.002	*C*. *jejuni*	8	19	3	1	4	Chicken	86%
0923.002.001	*C*. *jejuni*	8	2	18	14	40	Chicken	72%
0117.001.007	*C*. *jejuni*	7	1	73	43	180	Chicken	100%
0114.001.005	*C*. *jejuni*	7	0	62	29	188	Chicken	100%
0633.004.002	*C*. *coli*	6	9	7	3	17	Chicken	87%
0169.005.004	*C*. *jejuni*	6	2	74	133	30	Chicken	75%
0795.003.003	*C*. *jejuni*	6	0	63	51	153	Chicken	90%
0173.002.004	*C*. *jejuni*	5	6	24	16	19	Chicken	81%
0957.004.001	*C*. *jejuni*	5	0	58	36	154	Chicken	97%
0609.003.002	*C*. *coli*	5	0	94	143	165	Chicken	56%
0018.001.004	*C*. *jejuni*	4	0	77	56	114	Chicken	100%
0169.001.002	*C*. *jejuni*	3	9	1	10	1	Cow	71%
0609.006.004	*C*. *coli*	3	2	17	94	36	Cow	91%

^1^ Ranking based on Canadian *Campylobacter* Comparative Genomic Fingerprinting database (C3GFdb) which, at the time of this writing comprised data on 21,232 *Campylobacter* isolates (accessed 2018).

^2^ Non-human source specificity was defined by determining the most prevalent non-human source associated with the CGF subtype and calculating the proportion of isolates from that source as a proportion of total isolates from non-human sources in the C3GFdb

**Fig 2:**
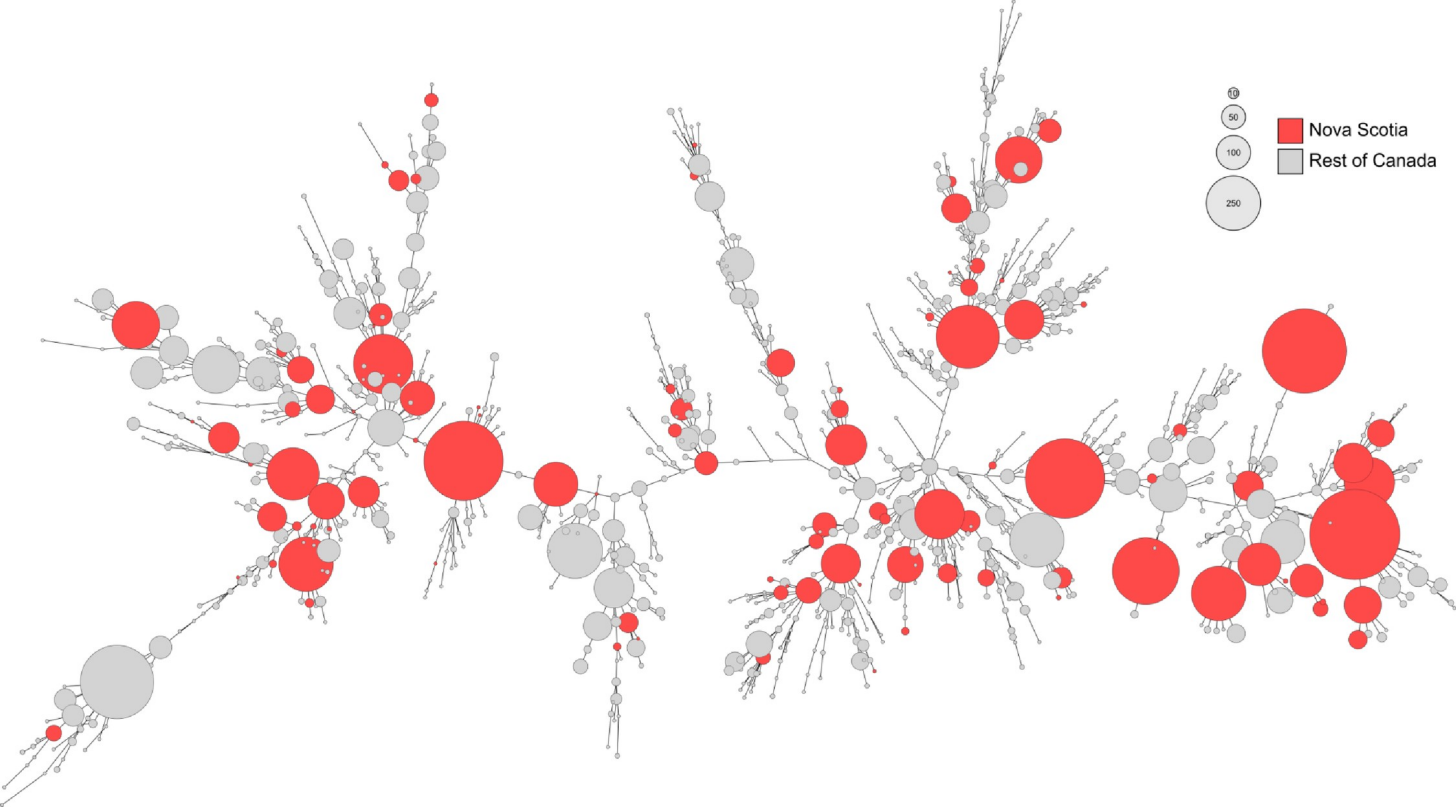
Population structure of Canadian chicken-associated *C*. *jejuni*. A minimum spanning tree of all CGF subtypes found in the C3GFdb which have been isolated from chicken; red circles represent subtypes from both Canada and this study, grey circles represent subtypes which were not recovered from chicken in this study. Node sizes are scaled based on the aggregate number of isolates in this study and the C3GFdb.

A comparison of Nova Scotia chicken subtypes observed in this study (n = 99) to all chicken subtypes in the C3GFdb (n = 1,057) showed that isolates from this study represented a genetically diverse collection representative of predominant subtypes (Table 2) and major genetic lineages circulating in the Canadian chicken production chain (Fig 2). The 99 subtypes observed in this study account for 3,836 of 6,821 (56.2%) of chicken isolates in the C3GFdb.

### Comparison of Nova Scotia chicken subtypes to those recovered from campylobacteriosis cases in Nova Scotia and across Canada

**Fig 3:**
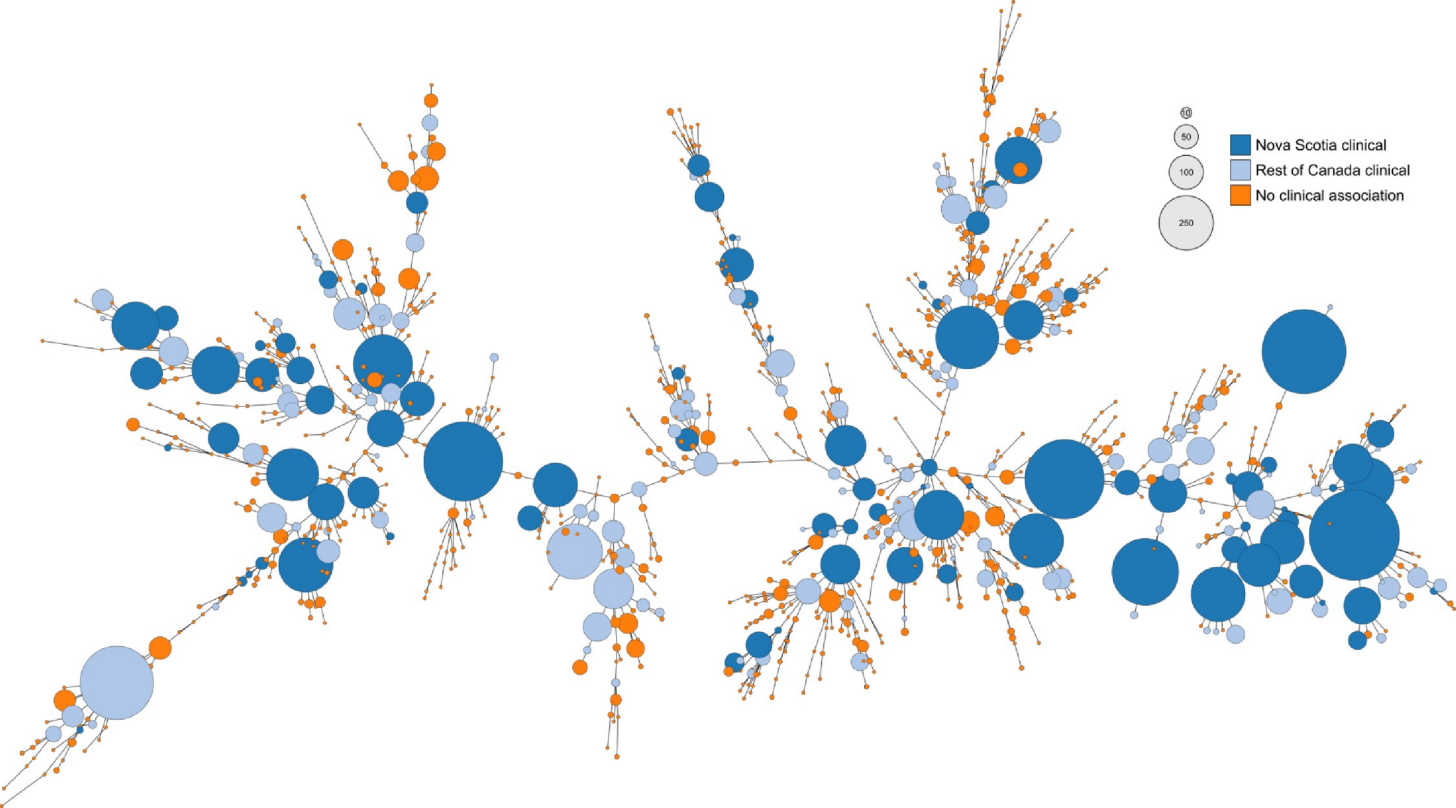
Distribution of human clinical association on the population structure of chicken CGF subtypes observed in Canada. A minimum spanning tree of all CGF subtypes found in the C3GFdb that have been recovered from chicken and their association with human clinical isolates. Represented are chicken subtypes observed in clinical cases from both Canada and this study (dark blue), in Canada but not in this study (light blue), and chicken subtypes not associated with a clinical case (orange). The size of each node is representative of the aggregate number of isolates in this study and the C3GFdb.

A comparison of isolates recovered from chicken as part of this study (n = 292; 99 subtypes) to contemporary human clinical isolates from Nova Scotia (n = 354; 152 subtypes) showed that only 36 subtypes had matches in both chicken and human isolates. However, nearly half (n = 171/354) of the human clinical isolates in this study belonged to these overlapping subtypes. Moreover, these 36 subtypes represent predominant subtypes circulating in the Canadian poultry chain that have been implicated in human clinical cases across Canada (Fig 3), including 47.9% (n = 3,270/6,821) of chicken and 30.1% (n = 1,406/4,669) of human clinical isolates in the C3GFdb. By contrast, the 63 Nova Scotia chicken subtypes lacking matches among contemporary human isolates in the study represent only 8.2% (n = 561/6,821) of chicken and 1.3% (n = 62/4,669) of human clinical isolates in the C3GFdb.

### Source attribution of human cases of campylobacteriosis in Nova Scotia

**Fig 4:**
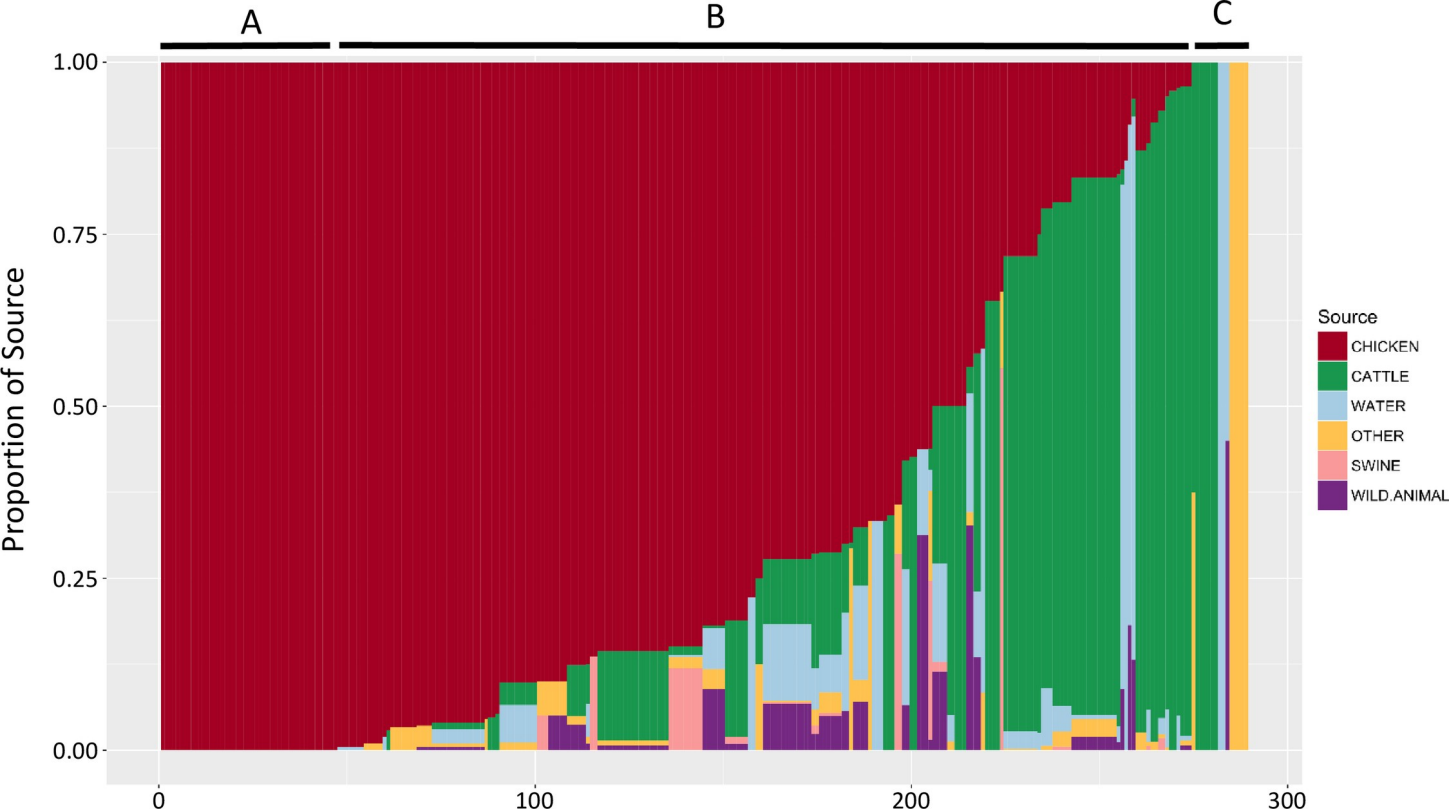
Source attribution for human campylobacteriosis cases in Nova Scotia. The relative contribution of non-human sources for each clinical subtype is displayed for 289 clinical cases based on the number of isolates in the C3GFdb. Source vectors have been clustered for visualization purposes.

Source attribution for Nova Scotia was investigated by examining CGF subtyping data for human clinical cases reported to Nova Scotia provincial public health laboratories between January 2012 and March 2015 (n = 354). Of 152 CGF subtypes observed among Nova Scotia *Campylobacter* human isolates in this study, 97 matched to subtypes containing isolates from non-human sources including chicken, cattle, swine, environmental water, wild animals and others. These subtypes, which represent 81.6% (n = 289/354) of human isolates (i.e. cases) in the study, were used for source attribution estimates. This included 22 subtypes (47 cases) observed exclusively in chicken (Fig 4A), 67 subtypes (227 cases) with varying degrees of chicken association (Fig 4B), and 8 subtypes (15 cases) that have yet to be observed in the Canadian chicken production chain (Fig 4C).

For each case, the subtype-based relative proportion of each non-human source is shown in Fig 4, where the overall area represented by each source represents an estimate of its relative contribution to human illness. Although 51.56% (n = 149/289) of clinical isolates had subtypes that were attributed to 4 or more sources, our results suggest that 67.7% of human campylobacteriosis cases in this study were attributable to chicken. Attribution estimates to non-chicken sources included cattle (20.7%), water (5.1%), wild birds and small mammals (2.2%), swine (1.1%), and other sources (3.2%).

**Fig 5:**
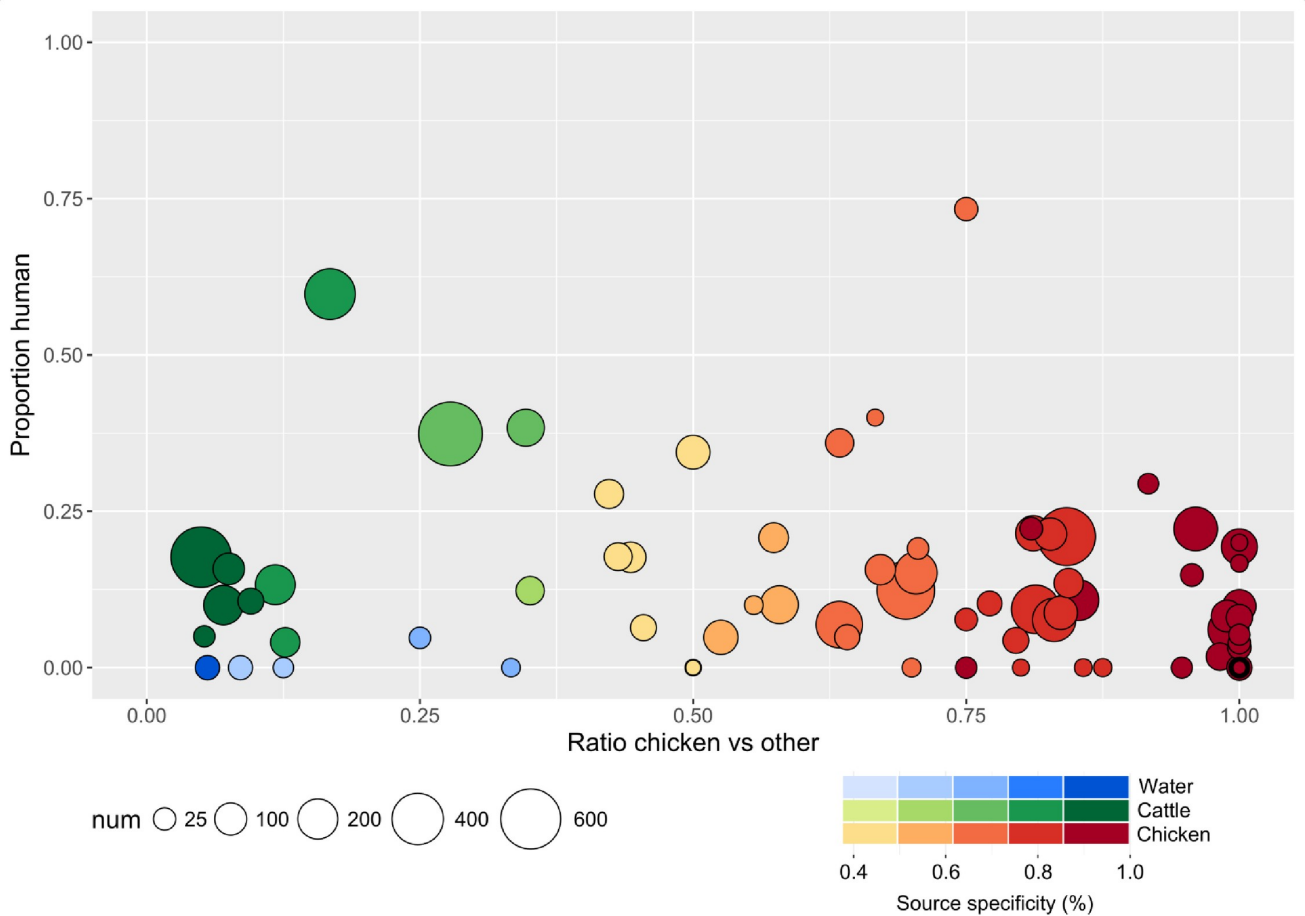
Degree of non-human and human source association among chicken subtypes observed in Nova Scotia. Along the x-axis, we show the degree of chicken association, expressed as the ratio of the number of isolates from non-chicken sources (cattle, swine, water, wild, other) vs. the number of isolates from chicken. Along the y-axis, we show the level of human clinical association, where the number of clinical isolates is shown as a proportion of the total number of isolates in that subtype. The degree of shading is used to indicate the level of source specificity.

We investigated the host association of the 99 chicken subtypes observed in Nova Scotia as part of this study by examining the degree of source association of each subtype in the C3GFdb. Most of the chicken subtypes, including a majority of human/chicken overlapping subtypes (n = 28/36), were primarily associated with chicken and show varying degrees of prevalence and human clinical association (Fig 5). A small number of subtypes primarily associated with non-chicken sources included some that were primarily associated with cattle. These subtypes have been strongly implicated in human cases of campylobacteriosis across Canada, including a larger number of human clinical isolates in the C3GFdb (722 vs. 684 isolates) than the chicken-associated subtypes.

## Discussion

This study represents the largest sampling of *Campylobacter* on raw, retail poultry in Nova Scotia and amongst the largest of the Canadian studies to date [22,23,25,31,32]. Although several studies have previously examined prevalence of *Campylobacter* at the farm and abattoir level, very few studies actually report on the prevalence of *Campylobacter* on retail poultry. However, prevalence at the farm and abattoir level does not necessarily correlate to the level of risk to the final consumer since it does not reflect the primary point of exposure [14]. This study found that 65% of the raw packaged chicken sold in Nova Scotia were contaminated with *Campylobacter* spp., consistent with some previously reported prevalence rates [33–35], in which 62% to 70% of raw retail chicken tested positive. In contrast, other Canadian studies have reported considerably lower prevalence rates of *Campylobacter*, ranging from 29% to 42.8% of the samples tested [22–24,36,37]. Excluding a subset of samples tested in two studies [22,23], none of these studies were conducted in Nova Scotia. The considerably lower prevalence rates of *Campylobacter* spp. reported by these studies may be largely due to differences in the methodology and sampling. The most notable differences between this study and others include that > 200 g samples were used, the type of enrichment broth, that no cycloheximide was added to the enrichment broth and that passive filtration was used to reduce the presence of competing background flora. These factors have been shown to affect the detection and isolation of *Campylobacter* [23,38,39]. The seasonality of this study (summer to early fall) is also likely to have resulted in increased recovery. Prevalence of *Campylobacter* is well known to increase in broiler chickens in the summer months [22,40,41]. The prevalence during the same time period in retail poultry analysed in the Canadian National Microbiological Study was 50%, indicating that methodology likely has a more important influence than seasonality [22]. While the prevalence rates vary between studies, there is agreement between this study and previous Canadian studies that *C*. *jejuni* is the most frequently isolated species on poultry (> 80%), followed by *C*. *coli* and then *C*. *lari*.

The CGF subtyping of *C*. *jejuni* and *C*. *coli* isolated from Nova Scotia retail chicken revealed 99 distinct subtypes. Many of these subtypes have been previously reported in Canada while others were novel (i.e. not previously observed in the Canadian *Campylobacter* CGF database (C3GFdb)). Overall, the most prevalent chicken CGF subtypes recovered during this study are in-line with the most prevalent subtypes found nationally (Fig 2). Of the 20 most abundant subtypes in this study, 12 were also among the 20 most prevalent subtypes in chicken in the C3GFdb (Table 2) and all but two (0169.001.002 and 0609.006.004) were strongly associated with chicken as their primary animal source, which is consistent with chicken production systems that are minimally impacted by other known reservoirs of *Campylobacter*, such as cattle and other livestock. Recovery of abundant chicken subtypes spanned multiple weeks, meat establishments and retail stores, and represented a significant proportion of the isolates recovered during this study. The ability to recover subtypes over weeks and months illustrates the ubiquitous nature of these subtypes or the ability of some *Campylobacter* to persist for long periods of time at the farm and in processing environments, leading to repeated contamination of flocks and carcasses [42].

A comparison of CGF subtypes observed in Nova Scotia retail chicken and contemporary human clinical cases revealed subtypes that were specific to each group, as well as a small number of subtypes (36/215 subtypes) that were shared between humans and retail poultry. Despite the low proportion of overlapping subtypes, these included the most abundant chicken subtypes recovered in this study and accounted for nearly half of the NS human isolates. Moreover, because these subtypes represent nearly half of the chicken isolates and nearly a third of the human clinical isolates in the C3GFdb, they represent subtypes circulating in NS chicken that are the most concerning to human health. For example, subtypes 0253.004.001, 0926.002.001, and 0083.001.002 are all highly chicken-associated and rank highly among both chicken subtypes in the C3GFdb (ranks 1^st^, 2^nd^, and 5^th^, respectively) and subtypes isolated from humans (9^th^, 7^th^, and 4^th^, respectively). This would suggest that there are lineages in the *Campylobacter* population circulating in Canada that pose a higher risk to human health, likely through increased chicken-associated exposure leading to infection. Possible factors influencing the high recovery rate of these subtypes from retail poultry along with a high prevalence in human clinical cases compared to other subtypes could be differences in survival along the food chain and potential virulence of some *C*. *jejuni* strains [43].

Although several studies have demonstrated that the most abundant subtypes found on chicken are almost always observed in humans [32,44–46], we found subtypes with moderate prevalence in chicken that were rarely associated with human illness. These subtypes have been observed almost exclusively in chicken (> 90% non-human source specificity) and although the impact of these subtypes on human health may not be fully understood at this time, the data suggests that such subtypes are only occasionally implicated in human illness. The lack of observed human clinical cases could be due to the inability of these subtypes to survive prolonged storage conditions (e.g. at the retail level), strong host specificity (i.e. more adapted to chicken than humans) or reduced virulence potential compared to other subtypes [43]. Overall, these subtypes can represent a population of *Campylobacter* in which there is a risk of human exposure but not necessarily an important risk to human health.

Source attribution based on an examination of non-human source association among CGF subtypes observed in Nova Scotia clinical isolates showed highest attribution to chicken (67.7%). This estimate is remarkably consistent with that of a recent study by FoodNet Canada in the province of Ontario [14] despite significant differences in methodology. Whereas Ravel et al. used an exposure-based Hald model using local non-human data, we used a basic Dutch attribution model but included source data from across Canada, which allowed us to generate attribution estimates on the 81% of human isolates with subtypes matching non-human isolates in the C3GFdb. Interestingly, the second most prominent attribution estimate in our study was cattle (20.7%) also consistent with the estimate by Ravel and colleagues [14], which attributed 14% of cases to cattle manure exposure. Importantly, we observed several subtypes in Nova Scotia chicken implicated in human clinical cases for which the primary association was cattle. Subtype 0169.001.002 for example, is both the most abundant subtype in the C3GFdb and one that has been associated with the largest number of human clinical cases. Although this subtype is observed in chicken, it has a much stronger association with cattle (> 71%), which appears to be its primary reservoir. This subtype forms part of the ST-21 clonal complex group, which has also been described as having a host ecology primarily associated with cattle and chicken [45,47], consistent with the possible transmission from cattle to chicken flocks. Thus, while the most common transmission route to humans for these strains likely remains contact with contaminated chicken products, the ability of strains from these genetic lineages to colonize multiple hosts likely enhances the probability of propagation to chicken, highlighting the need to examine the potential role of non-chicken sources in the epidemiology of campylobacteriosis through contamination of the chicken supply chain.

## Conclusion

The high degree of correlation between the Nova Scotia and Canadian prevalence rates and comparable abundance in subtype profiles provide evidence that this study conducted in Nova Scotia is reflective of the *Campylobacter* population circulating at the national level. While the commonality of a subtype between human clinical cases and chicken does not correlate directly to causation, it does provide evidence that retail chicken act as a significant source for *Campylobacter* subtypes known to be implicated in human infections in Nova Scotia. The application of whole-genome sequencing analysis to examine the molecular epidemiology of campylobacteriosis in Nova Scotia is likely to shed light on the direct link between *C*. *jejuni* strains circulating in poultry and those observed in human clinical cases [48]. Future work should also focus on investigating factors (e.g. differences in virulence, ability to survive in the food production systems, or in their ability to colonize a range of hosts) that influence subtype prevalence in chickens and the degree of risk to humans.

## Supporting information

S1 Appendix Isolate information. (XLSX)
